# TreeMap, a tree-level model of conterminous US forests circa 2014 produced by imputation of FIA plot data

**DOI:** 10.1038/s41597-020-00782-x

**Published:** 2021-01-15

**Authors:** Karin L. Riley, Isaac C. Grenfell, Mark A. Finney, Jason M. Wiener

**Affiliations:** 1 Missoula Fire Sciences Laboratory, Rocky Mountain Research Station, U.S. Forest Service, 5775 Highway 10 West, Missoula, Montana 59812 USA; 2 University of Montana, Marketing and Management Department, 32 Campus Dr., Missoula, Montana 59812 USA

**Keywords:** Environmental sciences, Ecological modelling, Forestry

## Abstract

A 30 × 30m-resolution gridded dataset of forest plot identifiers was developed for the conterminous United States (CONUS) using a random forests machine-learning imputation approach. Forest plots from the US Forest Service Forest Inventory and Analysis program (FIA) were imputed to gridded c2014 landscape data provided by the LANDFIRE project using topographic, biophysical, and disturbance variables. The output consisted of a raster map of plot identifiers. From the plot identifiers, users of the dataset can link to a number of tree- and plot-level attributes stored in the accompanying tables and in the publicly available FIA DataMart, and then produce maps of any of these attributes, including number of trees per acre, tree species, and forest type. Of 67,141 FIA plots available, 62,758 of these (93.5%) were utilized at least once in imputation to 2,841,601,981 forested pixels in CONUS. Continuous high-resolution forest structure data at a national scale will be invaluable for analyzing carbon dynamics, habitat distributions, and fire effects.

## Abstract (summary)

**Table:** 

Measurement(s)	forested area
Technology Type(s)	machine learning
Factor Type(s)	biophysical variables • geographic location • forest type • forest height • forest percent cover
Sample Characteristic - Environment	vegetation layer
Sample Characteristic - Location	United States of America

Machine-accessible metadata file describing the reported data: 10.6084/m9.figshare.13285904

## Background & Summary

Forest data at the level of individual trees are used for a wide variety of applications, including estimation of forest biomass^1^ and terrestrial carbon^2^, as well as more specialized projects such as modelling the risk of wildland fire to carbon resources^3^, our ultimate objective. Detailed forest inventories are conducted routinely across the forested areas of the US as part of the US Forest Service’s Forest Inventory and Analysis program (FIA). The FIA databases contain tree-level information from thousands of 672m^2^ plots that are systematically located and periodically remeasured^4^. However, the sampled plots are widely separated (at a density of approximately one plot per 24.3 km^2^) and thus do not provide wall-to-wall coverage of forest information that is critical to regional- or continental-scale modeling and analysis^5^. This paper reports on the development of a mapping methodology and data product containing continuous forest inventory data imputed at 30 × 30m resolution from the FIA sample plots across the continental US (CONUS).

**Fig. 1:**
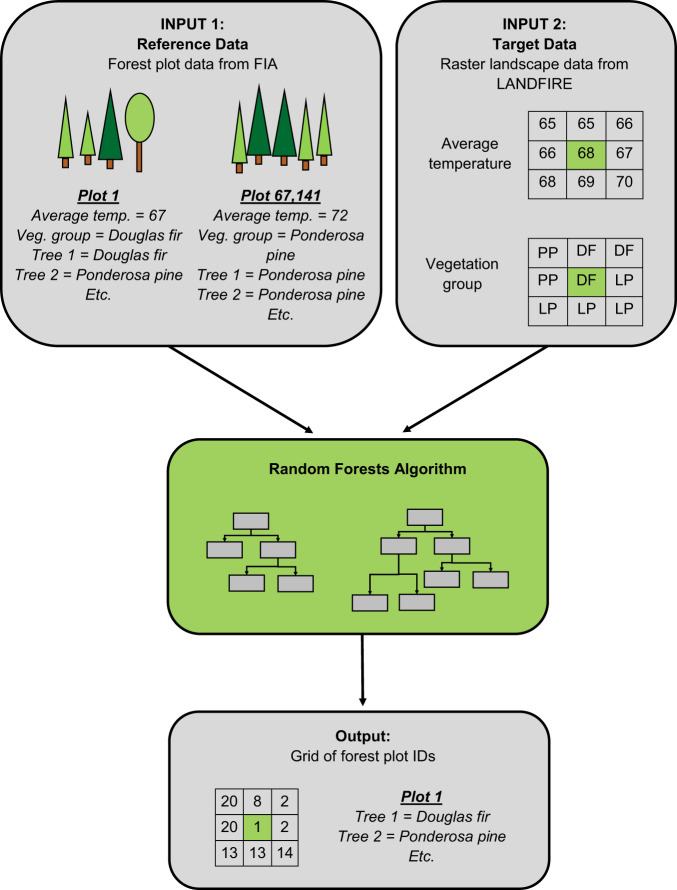
The project workflow. FIA forest plots (reference data) and raster landscape data from LANDFIRE (target data) were employed in a random forests algorithm that imputed the plot data to all forested pixels on the CONUS-wide landscape.

The FIA plot identifiers were imputed at 30 × 30m resolution for all CONUS forests using a machine-learning approach called random forests (Fig. 1). Random forests is being used increasingly in the ecological sciences^6–8^ because of the many advantages it offers; for example, 1) the user does not need to specify the form of relationships among variables (as is required in regression and some other techniques), and 2) it can employ both continuous and categorical variables^6^. These two advantages were pertinent to this project, in which interrelationships amongst the variables may be complex and non-linear, and two variables were categorical^9^. Due to the non-linear and categorical nature of the input variables, most other approaches including gradient nearest neighbour (GNN) were precluded^9^.

The random forests method requires two data sets: 1) *reference data*, comprised of detailed observations at selected sparse locations across the landscape, and 2*) target data*, a set of less detailed observations available for the entire landscape^10^. Random forests then uses a set of predictor and response variables specified by the user to select the best match from the reference data and impute it to each pixel of target data^10^. For reference data, we used field measurements of forest plots by the U.S. Forest Service’s Forest Inventory and Analysis program (FIA)^4,11^. For target data, we used raster data at 30 × 30 meter spatial resolution provided by the LANDFIRE project^12,13^. Though other sources of forest plot and landscape data exist, we chose the LANDFIRE data because they are compatible with a set of fire risk simulations for the US^14^, enabling us to conduct analysis of carbon risk in the future, and we chose the FIA forest plot data because they are nationally consistent and have stringent quality control. Both the LANDFIRE and FIA data are publicly available, another requirement for this study. The output is a raster of IDs of the best-matching plots at 30 × 30m spatial resolution (Fig. 1); we name this output dataset TreeMap 2014.

In an earlier project, we used a similar method to impute FIA data to LANDFIRE c2008 rasters for forests of the western U.S^9^. LANDFIRE provides mapping on a 2-year return interval; here we update the previous work to the year 2014 and extend the spatial extent across CONUS, thereby increasing the number of source plots available for imputation and raising the accuracy of the dataset. In addition to the set of location, topographic, and biophysical variables^9^, we added two new variables to capture recent disturbances to the forest.

The c2008 dataset^15^ is currently being used by land managers to inventory habitat types at regional scales (Daniel Couch, personal communication 10/29/2018), and by researchers to evaluate tradeoffs between fuel management and timber harvest targets^16^ and model hydrologic effects of fuel treatment (Nicholas Povak, personal communication, 10/29/2019). The three response variables (forest cover, height, and vegetation group) were chosen to serve as estimators of forest carbon at risk from impact by wildfire, one of our primary goals for developing these data. Other potential future uses include 1) modelling effects of surface roughness on wind, 2) modeling fuel treatment effect on future vegetation and fire risk, and 3) species envelope modelling, among others.

## Methods

### Disturbance analysis

Methods used for random forests imputation in the previous edition of this dataset^9^ did not incorporate any variables related to disturbances on the landscape. Consequently, the dataset may have underestimated dead trees in recently disturbed areas and limited its utility, for example, in estimating hazard to wildland firefighters from falling snags^17^, habitat for cavity-nesting birds, and potential coarse woody fuel sources. We therefore conducted an analysis to address this concern, since based on the results, variables could be added to the methodology of the new c2014 dataset to better capture the effects of disturbance on forest structure.

To this end, the number of dead trees in the reference (FIA plot) data was estimated from the TREE table within the FIA database via the STATUSCD field that represents the condition of each tree: 1 = live tree, 2 = dead tree, and 3 = cut and removed. Further, the TREE table indicates whether dead trees qualify as “standing dead” via the STANDING_DEAD_CD field; to qualify as standing dead, the tree must be at least 5” DBH, have a bole that has an unbroken length of at least 4.5 feet, and lean less than 45 degrees from vertical. With these fields, we calculated for each plot the number of standing dead trees (STANDING_DEAD_CD = 1). We found that disturbance by fire and insect/disease increased the number of dead trees on a plot; the effect was statistically significant for fire. Other disturbances (e.g. timber harvest) did not increase the number of dead trees on a plot. Therefore, we retained only fire and insect/disease as disturbance categories, as recorded by FIA in the CONDITION table via the fields DSTRBCD1, DSTRBCD2, DSTRBCD3 (Disturbance Code 1, 2, and 3) and DSTRBYR1, DSTRBYR2, and DSTRBYR3 (Disturbance Year 1, 2, and 3)^4^. If a plot was disturbed by either of these causes more than once during the period of record, we assigned the most recent disturbance, with any disturbance by fire being recorded preferentially over insect/disease.

To determine which pixels of target data were affected by disturbance, we combined LANDFIRE’s annual disturbance grids to make a single grid of all pixels that were disturbed between 1999 (the first year the grids were created) up to 2008 (the year of the first tree list dataset). We tracked only disturbance types that were expected to have an impact on the number of dead trees; therefore we tracked only fire, insect and disease and unknown disturbances, and not timber harvest or herbicide application. If a pixel was disturbed more than once, we used the same logic we applied to the forest plot data, assigning the most recent disturbance, with any disturbance by fire being preferentially recorded over insect/disease.

We found that the c2008 dataset underpredicted dead trees in burned areas, based on a two-sided Kolmogorov-Smirnov test (p-value = 1.2^−13^, *n* of burned plots = 356, *n* of undisturbed plots = 12,939). We therefore added a categorical class variable that tracked disturbance status (burned, insect/disease, or undisturbed) and a second numeric variable that tracked time since disturbance, since stand dynamics would be expected to change over time, as some dead trees fall.

### Data sources

Two sets of data are required for random forest imputation: 1) *the reference data* are detailed measurements at specific points on the landscape (for this project, the reference data are measurements from FIA forest plots), and 2) *the target data*, or spatial data for the entire landscape, to which the reference data are imputed (in this case, the target data comes from satellite-derived vegetation, topography, and biophysical grids produced by the LANDFIRE project^12,13^). The suite of predictor and response variables must be available in its entirety for both the reference and target data. In essence, the random forests methodology takes detailed measurements that are available only at sparse locations and assigns them to maps of more generalized landscape characteristics, in order to model detailed measurements continuously across the landscape.

### Target data

LANDFIRE gridded data contributed three vegetation variables (Existing Vegetation Cover (EVC), Existing Vegetation Height (EVH), and Existing Vegetation Group (EVG)), three topographic variables (slope, aspect, and elevation), and two disturbance variables (disturbance type and year) (Table 1). In the LANDFIRE target data, spatial mapping of vegetation type is done under the raster layer called Existing Vegetation Type^13^, which maps the existing locations of an ecological systems classification;^18^ from the EVT raster we drew the broader Existing Vegetation Group (EVG) for this analysis. The EVG is a broader classification into which several EVTs are grouped. A total of 76 EVGs were present in CONUS (Online-Only Table 1). We obtained a suite of six biophysical variables in raster format by direct request from LANDFIRE: photosynthetically active radiation, precipitation, relative humidity, maximum temperature, minimum temperature and vapour pressure deficit^12,19^. These grids were generated by the biophysical simulation model WxBCG, and estimate average annual weather^12^. All grids were at 30 × 30m spatial resolution and for CONUS extents. As part of this submission, we obtained permission from LANDFIRE to store these grids on figshare under the dataset name, “LANDFIRE Biophysical Gradient Raster Datasets”^20^.

**Table 1:** Target data sources and versions.

Category	Data layer/variable name	Data URL or source	Data version
Vegetation	Existing Vegetation Cover (EVC)	www.landfire.gov/version_comparison.php?mosaic = Y	1.4.0 (c2014)
	Existing Vegetation Height (EVH)	“	“
	Existing Vegetation Group (EVG) derived from the Existing Vegetation Type (EVT) raster	“	“
Topography	Aspect	“	1.2.0
	Slope	“	“
	Elevation	“	“
Biophysical	Photosynthetically active radiation	On figshare under the name, “LANDFIRE Biophysical Gradient Raster Dataset”^20^	NA
	Precipitation	“	“
	Relative humidity	“	“
	Maximum temperature	“	“
	Minimum temperature	“	“
	Vapor pressure deficit	“	“
Disturbance	Disturbance code (DistCode)	www.landfire.gov/version_comparison.php?mosaic=Y	Annual grids from 1999–2014 including both disturbance type and year
	Disturbance year (DistYear)	“	“
Location	Latitude and longitude	Obtained by calculating the centroid of each 30x30m pixel in the raster	Any LANDFIRE version since the grid remains the same across versions

We ran the imputation for forested areas in the continental US (CONUS), defined as those having greater than 10% tree cover. This definition is used by FIA to demarcate forested versus non-forested areas (however, if a plot that was historically forested was disturbed and is temporarily below 10% cover but expected to recover, FIA may still consider a plot to be forested; we omitted any FIA plots or pixels below 10% from the imputation for consistency). In order to limit the rasters to pixels with forest cover, we subset the EVC raster to those classified as forest with 10% or greater cover. We used this subset of pixels to then mask the EVT raster, and discarded pixels classified as developed or agricultural forest such as orchards or urban forest. Because vegetation type is categorical, random forests could not perform the imputation unless each EVG had at least one forested plot keyed to it (see Reference Data subsection below for description of methodology for keying plots to EVG). Six EVGs were present in our forest mask that had no plots keyed to them, and the following substitutions were performed: 1) we eliminated 2,483 pixels that had EVG 649 (Tallgrass Prairie) which were within the forest mask, 2) we recoded 606 pixels of EVG 692 (Spruce Flats and Barrens) to EVG 693 (Spruce-Fir-Hardwood Forest) as this EVG was similar in spatial location and species type, 3) we recoded EVG 705 (Introduced Wetland Vegetation) to EVG 668 (Eastern Small Stream Riparian Forests), as this EVG was similar in spatial location and description, 4) we omitted EVG 730 (Transitional Forest Vegetation) since it signifies recently logged or severely disturbed areas unlikely to have substantial forest cover, and 5) we manually recoded some plots to Loblolly Pine Forest and Woodland (EVG 697) and Loblolly Pine and Loblolly Pine-Slash Pine Forest and Woodland (EVG 698) (regarding these two EVGs, see Reference Data subsection below for more detail on methods). The final forest mask had 2,841,601,981 forested pixels.

LANDFIRE bins forest EVC into nine categories: 10–20%, 20–30%, 30–40%, 40–50%, 50–60%, 60–70%, 70–80%, 80–90%, and 90–100% cover. We assigned each pixel the midpoint of the bin it represents (e.g. 15, 25, 35, 45, 55, 65, 75, 85, and 95%).

Similarly, EVH is binned into five categories in the LANDFIRE raster, which we reclassified to a single value for each bin at its midpoint: specifically, 0–5 m reclassified to 3, 5–10 m reclassified to 8, 10–25 m reclassified to 18, 25–50 m reclassified to 38, and >50 m reclassified to 70 (the tallest plot in our database was 89 m, so this corresponded to the arithmetic mean of the bin). EVH corresponds to the height of the dominant vegetation.

We recoded LANDFIRE’s annual disturbance layers, 1) limiting them to only two disturbance types (fire and insect/disease), and 2) retaining only the most recent disturbance type and year between 1999–2014, with fire taking precedence over insect/disease, since fire was the only disturbance type that had a statistically significant effect on prediction of tree mortality. In other words, if a pixel burned at any time during 1999–2014, it was assigned that disturbance code, even if it was affected by a more recent insect/disease infestation. If the pixel was affected only by insect/disease and never by fire during 1999–2014, it was assigned the code and year of the insect/disease infestation.

In order to obtain the location of each pixel, we calculated the latitude and longitude of each pixel’s centroid.

### Reference data

**Fig. 2:**
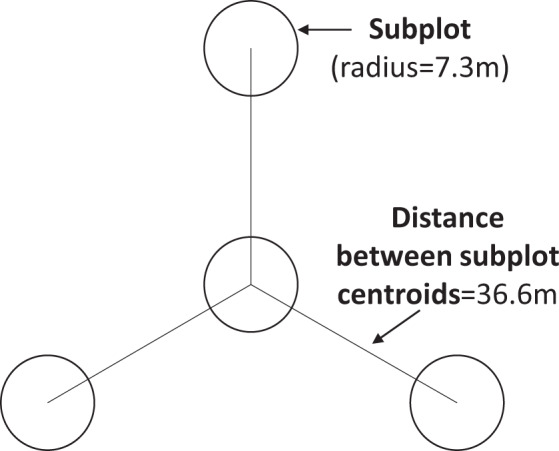
The design of FIA forest plots includes four subplots where trees larger than 12.7 cm at breast height are measured^4^. Each subplot is 7.3 m in radius with three subplots arranged at 120-degree spacing around a center subplot, with a separation distance between the subplot centers of 36.6 m^4^.

We obtained Forest Inventory Analysis (FIA) data version 1.7 by state for all 50 states from the online and publicly available FIA Data Mart on 10/31/2017^11^, using the attributes for aspect, slope, and elevation as well as individual tree diameter, height, species, status (dead or alive), and disturbance history (Online-only Table 2). The tree-level attributes were not used as variables in the random forests model but were necessary for various applications of the output TreeMap database. FIA measures plot-level and tree-level attributes on a regular spatial and temporal interval in all 50 states using a standardized plot design^4^ (Fig. 2). If a plot spans different land use or vegetation conditions (e.g. harvesting or recent fire affected only part of the plot), FIA flags this plot as multi-condition^4^; we endeavored to use only single-condition plots in the imputation so that they would be more or less homogenous. Of the 67,141 plots we obtained from FIA, 65,652 (97.8%) were single-condition and 1489 (2.2%) were multi-condition. We obtained a second set of 2,319 multi-condition plots inventoried in 2014; we used this second set of plots in model validation.

We obtained several variables needed for the imputation directly from FIA’s online database: aspect, slope, elevation, disturbance type, and disturbance year (Online-only Table 2). FIA makes public only the approximate location of plots because of a provision in the Food Security Act of 1985^4^. Plot locations are “fuzzed” within a 1-mile radius (random error added to the true plot coordinates) and plot locations on private land are swapped with other plots in up to 20% of cases^4^. A Memorandum of Cooperation with FIA permitted us to use the true plot coordinates (not fuzzed) as location variables and for extracting biophysical variables from the LANDFIRE grids (Online-only Table 2).

We used the Forest Vegetation Simulator (FVS) to calculate plot-level forest cover and height from the tree records^21,22^. Plot locations were overlaid with a geospatial layer of geographical FVS variants that contain different equations for allometry (including crown radius) and growth. Several hundred plots fell outside the FVS variant shapefile boundaries by short distances, appearing in bodies of water or Canada or Mexico; these were manually assigned to the closest variant as they were more likely to be the result of inaccuracies in the variant shapefile than locations measured on the ground at FIA plots. We then calculated cover and height via FVS’s “StrClass” keyword^22^. Cover is calculated by estimating the percent of ground area covered directly by the crowns of trees, and corrects for crown overlap^22^. While cover figures calculated this way are continuous, we rounded them to the midpoint of the nearest 10% cover bin to match the ordinal classification in the LANDFIRE EVC raster. We refer to these bins by using the midpoint in the remainder of this manuscript. In order to calculate a single plot-level height value from the individual tree records, the FVS “StrClass” routine divides the forest canopy into different strata, when appropriate, via gaps in the distribution of tree heights. We assigned to each forest plot the height of the top stratum, which was computed by averaging the height of the nine trees centered around the 70^th^ percentile tree^22^. Height values calculated this way were also continuous, and we reclassified them to match the midpoint of the nearest of the five bins used in the LANDFIRE EVH map (i.e 3, 8, 18, 38, and 70m).

FIA plots were keyed to the same set of Ecological Systems appearing in the EVT raster^18^ using LANDFIRE’s keying system^23^. The keying system, or “AutoKey”, uses a ruleset for each of 16 regions in the U.S. Inputs to the AutoKey include: 1) the basal area by live tree species, 2) for plots in the Western U.S., where understory data is recorded by FIA, cover of understory species, 3) a list of seedling species, 4) the isobioclimate^24^, 5) the elevation, 6) the ecological subsection^25^, 7) the EPA ecoregion^26,27^, and 8) landform (e.g. flat plains, hills)^18,28^. This data is first run through the “ruderal” classification system; if a plot receives a ruderal classification, then that is the final classification; otherwise the plot is run through the ecological systems key. The AutoKey outputs an EVT, which we then reclassified to the broader EVG. We found that no plots had classified to the Loblolly Pine Forest and Woodland (EVG 697) or Loblolly Pine and Loblolly Pine-Slash Pine Forest and Woodland (EVG 698) EVGs despite the presence of plots stocked with loblolly pine and slash pine; instead plots stocked with these tree types had all classified to ruderal types. We manually identified a subset of plots composed of loblolly and reassigned their EVGs, with 44 plots fitting the description of Loblolly Pine Forest and Woodland (EVG 697) and 24 fitting the description of Loblolly Pine-Slash Pine Forest and Woodland (EVG 698).

### Random forests imputation

Random forests leverages a “forest” of decision trees to make predictions^6^ (Fig. 1). Here, we used the yaImpute package in R to run a modified random forests imputation^9,10,29^. The reference data (from observed FIA plots) were used in random forests to construct 249 decision trees per LANDFIRE zone, 83 for each of the response variables (Existing Vegetation Cover (EVC), Existing Vegetation Height (EVH), and Existing Vegetation Group (EVG)). Previous experience with 500 trees showed only slight improvement in accuracy at great computational burden^9^.

In the construction of each tree in the random forest, 66% of the observations (FIA plots in this case) are randomly chosen, with the rest of the observations being used to calculate an out-of-bag error rate^6^. Out-of-bag error rates for this project were low, ranging from 0.003164–0.023375 for EVC, 0.000151–0.002316 for EVH, and 0.000000–0.006213 for EVG, depending on the zone. As mentioned above, a requirement of random forests is that the same suite of predictor and response variables must be present for both the target data and the reference data. The predictor and response variables are used to make binary partitions in the data during the construction of each decision tree. For a given tree, the data start in a single group or “bucket”, then at the first node, several predictor variables are randomly chosen for assessment (the number chosen is the square root of the total number available, so if nine predictor variables are used in the imputation, then three would be chosen at each node). The predictor variables are assessed to determine which reduces the variance in the response variable the most, then that variable is selected and applied in the binary partitioning of the data (or splitting of the data into two groups or buckets). Binary partitioning of each tree continues until the variance in the data can’t be significantly reduced or partitioning would result in fewer than five observations in a bucket^6^.

Note that in this application of random forests, where several response variables can be used at once, the response variable isn’t a response variable in a traditional sense, since what we endeavour to predict for each landscape target pixel is the ID of the best-matching plot and not a value for the response variable itself. The predictor and response variables are used to find associations among the reference data, thus identifying which forest plots are most like each other.

**Fig. 3:**
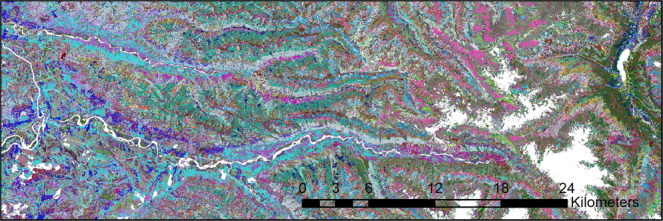
Aerial view of tree list output for a section of the Olympic Peninsula, Washington. Each plot ID appears with a different color. Plots cluster along biophysical gradients driven by mountainous topography and stream corridors.

In order to make predictions for a given pixel of the target data, the values of the predictor and response variables at that pixel are run through each decision tree, and the IDs of the plots that appear in the terminal bucket with it are recorded^6,9^. The forest plot that is assigned to the pixel is the one that appears most frequently with it in the 249 terminal buckets – hence the ID of that forest plot is assigned to the pixel (with any ties split randomly)^6,9^. Once all pixels in the landscape have been run through random forests, the result is a raster grid of best-matching FIA forest plot IDs at 30 × 30m spatial resolution (Figs. 1 and 3). The plot IDs can be tied back to the FIA database in order to map many different attributes, including number of live and dead trees, species presence/absence, etc.

**Fig. 4:**
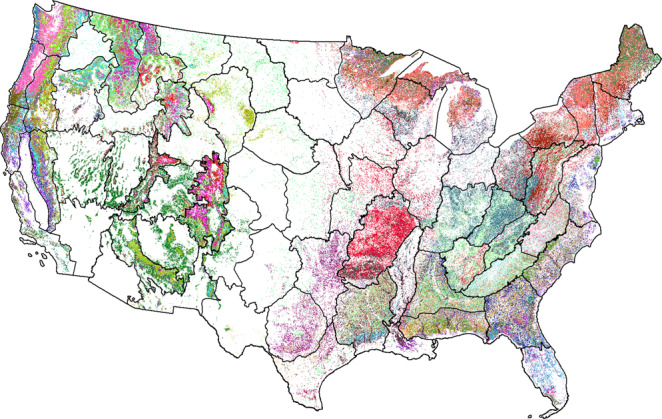
Map of LANDFIRE zones and forested pixels. Black outlines illustrate CONUS subdivided into LANDFIRE zones. Non-forested pixels (for which the imputation was not performed) are shown in white. Forested pixels for each EVG class are assigned a unique color.

The yaImpute package can process a maximum of 32 classes of data at a given time^10^. Because more than 32 EVGs were present at the national scale, we subdivided the data into LANDFIRE map zones (Fig. 4) and ran the imputation for one zone at a time. In running the imputation for each zone, we utilized only the FIA plots with EVGs that appeared in that zone.

## Data Records

The imputed forest data are stored in the US Department of Agriculture Research Data Archive^15^. The records consist of: 1) a grid of imputed plot IDs at 30 × 30m resolution in GeoTIFF format, 2) a table containing records for each tree on each plot in both.mdb and.txt format, and 3) metadata with details of data architecture and attributes. Many more plot characteristics can be accessed via the FIA DataMart^11^, which serves the data by state in a variety of formats including HTML, XLS, SQLITE, and CSV.

The LANDFIRE Biophysical Gradient Raster Datasets are shared via figshare with the permission of LANDFIRE^20^.

## Technical Validation

We found that 62,758 (93.5%) of 67,141 available plots were used at least once for imputation. In assessing the accuracy of the imputed dataset, there are several pertinent questions. The ultimate measure of agreement is how well the imputed dataset replicates conditions on the ground, which can be assessed at selected locations by comparing the attributes of the output grid of plot IDs to a set of recently measured FIA plots. Since the target LANDFIRE data were based on satellite imagery for the year 2014, the imputed dataset also has a vintage of 2014, and we used a subset of the FIA plots measured in 2014 to assess the accuracy of the imputed dataset (using FIA plots from previous years would not account for subsequent growth or disturbances between the time the plot was measured and 2014). We obtained the locations of 2,319 multi-condition FIA plots measured in 2014 and leveraged these in the validation of the imputed dataset. The accuracy of the imputed dataset depends heavily on the predictor variables that were drawn from the LANDFIRE target data; hence a second issue is how well the target LANDFIRE data itself compared to the conditions on the ground, which can be assessed using the same set of FIA plots from 2014. Errors and inaccuracies in the LANDFIRE target dataset will naturally propagate to the imputed dataset. Logically, then it also makes sense to compare the imputed dataset to the LANDFIRE target data on a pixel-by-pixel basis for all 2,841,601,981 pixels; if the methodology is performing well, then the values of forest cover (EVC), height (EVG), and vegetation group (EVG) derived from the imputed forest plots will correspond well with the values in the target LANDFIRE data. Agreement was measured in terms of the producer’s accuracy (probability that a category on the ground received that classification in the imputed map) and the user’s accuracy (probability that the imputed class in fact represents that class on the ground). In summary, validation was conducted to quantify the agreement between: 1) the plot conditions measured on the ground by FIA at the locations of the 2,319 multi-condition plots and the imputed dataset at these same locations, 2) the plot conditions at the locations of the 2,319 FIA plots and the LANDFIRE target data at these same locations, and 3) the LANDFIRE gridded target data and the imputed gridded data. Because we added disturbance code as a new predictor variable, we also assessed its accuracy by comparing the imputed grid and the LANDFIRE target data.

### Agreement between the FIA reference data and the imputed dataset

We checked for matches in three different attributes to quantify agreement between the gridded imputed dataset and the FIA reference data, at the locations of 2,319 multi-condition FIA plots measured in 2014: 1) the forest cover (EVC), 2) the forest height (EVH), and 3) the two tree species with the highest basal area. We chose the last measure in lieu of the EVG, as tree species are a direct and measurable characteristic of a forest plot and are not subject to any uncertainty in the vegetation group (EVG) categorization. The splayed footprint of a single FIA plot (Fig. 2) is 40.25 m in radius and spans several 30 × 30m pixels^4^. The combined area of the four subplots is 672 square meters, a figure relatively close to the size of a single pixel of 30 × 30m imagery (900 square meters). We checked all pixels whose centroid fell within the FIA plot radius for matches in these attributes (EVC, EVH, and tree species), since one or more of the four subplots may have fallen on these pixels. For each pixel within an FIA plot’s radius, we used the plot identifier number of the imputed plot to look up the corresponding values for EVC, EVH, and tree species. We recorded whether the EVC and EVH values of at least one pixel within a plot’s radius matched the value calculated for the plot. As another measure of imputation accuracy, we calculated whether the weighted cover value of pixels within the plot’s radius was within 10% of the plot value, and whether the weighted height value was within 5 m of the plot value. In order to evaluate whether the species composition was similar in the FIA and imputed data, we calculated the basal area of each live tree using the diameter (DIA field in the TREE table of the FIADB), which was then multiplied by the number of trees per acre (TPA_UNADJ field in the TREE table), and basal area was summed for each species on a plot using the species code (SPCD field in the TREE table). We then identified the species with the top two basal areas for each plot, and checked to see if any pixels within the plot footprint had at least one of the same top two species.

Of the 2,319 multi-condition plots obtained for the validation, 2,858 had at least one forested pixel within their plot radius (98.1%). Several possible reasons exist for this discrepancy, including that because the mask of forested pixels was derived from the LANDFIRE target data, there may be instances where a pixel is forested but LANDFIRE did not classify it as such or FIA may have measured trees on the plot but total cover may have fallen below the threshold of 10% cover. The cover bin of at least one pixel within a plot’s radius matched the plot value in 44.0% of cases, and the weighted cover value was within 10% of the plot value in 48.7% of cases (Table 2). The height bin of at least one pixel within a plot’s radius matched the plot value in 85.7% of cases, and the weighted height value of pixels within the plot’s radius was within 5 m of the plot value in 70.3% of cases. At least one of the two species with the highest basal area on the plot was also one of the top two species on at least one pixel within the plot’s radius in the imputed dataset in 76.7% of cases. While tree species itself was not either a predictor or response variable, the imputed dataset predicts tree species with a fairly high level of skill.

**Table 2:** Summary of accuracy assessment for TreeMap 2014. Accuracy is reported in percent followed in parenthesis by number of cases out of 2,858 FIA plots used in validation.

Response variable	At least one pixel within plot radius matches plot value in…	Imputed data	Weighted cover/height of pixels within plot radius is with 10%/5 m of plot value in…	Imputed data
	Target data (LANDFIRE)		Target data (LANDFIRE)	
Cover (EVC)	43.7% (1250)	44.0% (1257)	48.7% (1391)	48.7% (1393)
Height (EVH)	85.4% (2440)	85.7% (2449)	70.2% (2006)	70.3% (2010)
Tree species with two highest basal areas	NA	76.7% (2192)	NA	NA

### Agreement between the FIA reference data and the target LANDFIRE dataset

We repeated the analysis described in the section above using the target LANDFIRE dataset instead of the imputed dataset. The rates at which matches occurred were similar for EVC and EVH variables whether comparing the plot values to the LANDFIRE grids or to the imputed grid (Table 2). Specifically, the cover value of at least one pixel within a plot’s radius matched in 43.7% of cases (versus 44.0% in the imputed data), while the weighted cover value of pixels within the plot’s radius was within 10% of the plot value in 48.7% of cases (versus 48.7% of cases in the imputed data; Table 2). The height value of at least one pixel within the plot’s radius matched in 85.4% of cases (versus 85.7% of cases in the imputed data), while the weighted height value of pixels within the plot’s radius was within 5 m in 70.2% of cases (versus 70.3% of cases in the imputed data). It would appear that the accuracy of the imputed dataset in the cover and height categories is heavily driven by the target data, a question investigated more thoroughly in the next section.

### Agreement between the target LANDFIRE and imputed datasets

Here, we compared the gridded input LANDFIRE data and the gridded output imputed dataset on a pixel-to-pixel basis because both datasets are 30 × 30m grids. Since the target LANDFIRE data were used to generate the suite of predictor variables used to choose the best-matching plot for each pixel, if the random forests imputation performed well, the values of these variables should be similar in the imputed data.

From the raster of imputed plot IDs, we generated rasters based on the plot characteristics: one raster for cover (EVC), one for height (EVH), and one for vegetation group (EVG). Each of these rasters was then combined with the LANDFIRE target raster and the values compared on a pixel-by-pixel basis as a measure of imputation accuracy.

**Fig. 5:**
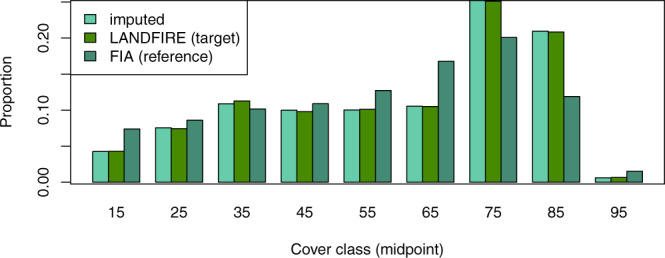
Proportion of FIA plots (reference data), LANDFIRE pixels (target data), and imputed pixels falling into each of the nine cover classes.

**Fig. 6:**
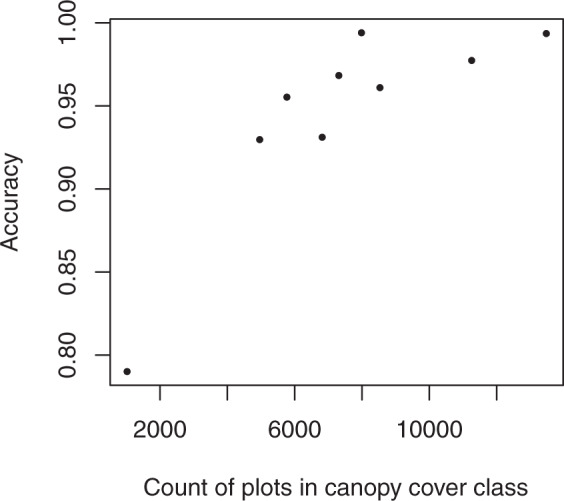
Producer’s accuracy by number of plots available for imputation in a cover class.

The forested mask of the LANDFIRE data included 2,841,601,981 pixels to which we imputed FIA plots. The imputed raster had the same forest cover class as the LANDFIRE cover raster on 97.2% of pixels. Agreement between the target and imputed data was above 92% for eight of nine cover bins, with the lowest producer’s accuracy at 79% for the 95% cover bin (Table 3). This bin had many fewer plots available for imputation (Figs. 5 and 6), thus it was more difficult for the random forests algorithm to match the cover values while simultaneously matching height and vegetation group (the other two response variables). Indeed, producer’s accuracy tended to increase with the number of plots available for imputation in a cover class (Fig. 6). The proportion of the landscape falling into each of the nine cover bins was similar across the three data sources (FIA plots, LANDFIRE data, and imputed dataset) (Fig. 5). However, the proportions were more similar between the imputed and target data than to the FIA plots. Since FIA plot locations are likely representative of the landscape as a whole, this suggests that LANDFIRE may have underestimated the number of pixels in the cover classes with midpoints of 15%, 55%, 65%, and 95%, and overestimated the number of pixels in the 75% and 85% cover classes (Fig. 5).

**Table 3:** Confusion matrix illustrating the number of pixels correctly and incorrectly classified in the various cover classes in the target and imputed datasets.

		Target									
		15	25	35	45	55	65	75	85	95	accuracy
**Imputed**	**15**	**113,259,693**	2,637,198	4,108,223	529,979	194,475	63,431	43,103	16,190	4,965	**0.937**
	**25**	5,017,812	**201,658,721**	6,422,551	788,100	355,138	131,744	62,121	16,986	4,713	**0.940**
	**35**	2,524,783	4,130,464	**297,997,650**	2,590,566	887,885	421,810	143,835	56,191	15,817	**0.965**
	**45**	565,367	1,312,429	7,681,977	**269,346,353**	3,154,093	1,437,401	346,507	89,705	18,245	**0.949**
	**55**	140,426	668,783	1,356,709	2,604,891	**276,169,764**	2,747,460	742,746	336,838	66,900	**0.970**
	**65**	214,322	366,572	1,160,443	807,754	2,940,943	**291,577,963**	1,771,381	671,736	145,536	**0.973**
	**75**	38,790	130,397	448,613	854,545	1,525,144	1,220,510	**709,268,338**	1,993,144	730,487	**0.990**
	**85**	43,800	115,488	663,493	485,575	902,553	573,635	1,144,017	**588,746,172**	2,918,495	**0.989**
	**95**	19,730	93,373	217,297	184,525	1,225,748	148,633	386,346	304,505	**14,691,240**	**0.851**
	**accuracy**	**0.930**	**0.955**	**0.931**	**0.968**	**0.961**	**0.977**	**0.994**	**0.994**	**0.790**	**0.972**

**Fig. 7:**
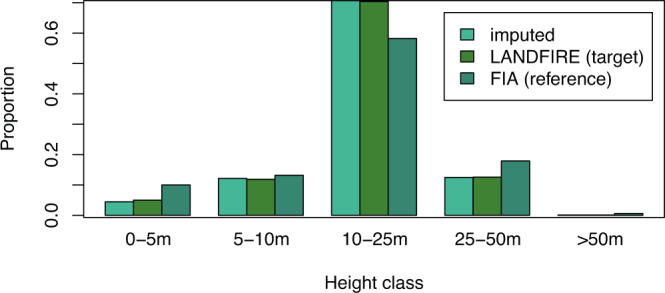
Proportion of FIA plots (reference), LANDFIRE pixels (target), and imputed pixels falling into each of the five height classes.

**Fig. 8:**
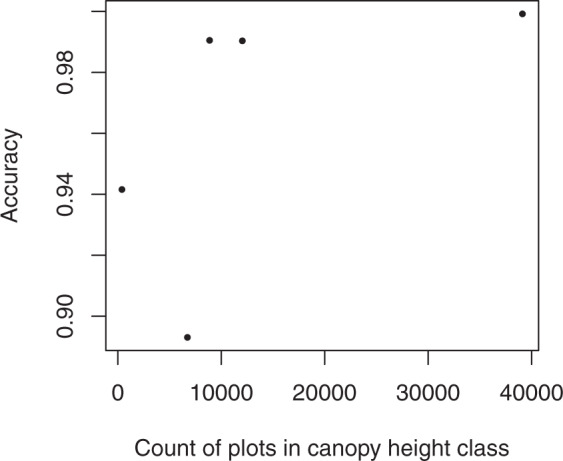
Producer’s accuracy by number of plots available for imputation in a height class.

Agreement between the imputed height values and the LANDFIRE target data was 99.2%. User’s and producer’s accuracies for height were over 94% in four out of five height classes, with producer’s accuracy of 89% in the 0–5 m height bin (Table 4). As with cover, accuracy tended to increase with the number of plots available for imputation in a bin; however, the producer’s accuracy for the 0–5 m class was lower than that of the 70 m class despite having more plots available for imputation (Figs. 7 and 8). The proportion of pixels/plots in each height bin was similar across the FIA plots, LANDFIRE data, and imputed dataset (Fig. 7); however, assuming that the FIA plots are indicative of proportions on the landscape, LANDFIRE appears to have underestimated the number of pixels in the 0–5 m and 25–50 m bins, and overestimated the number of pixels in the 10–25 m bin. Again, the proportions of the landscape in each height bin were more similar between the imputed and LANDFIRE datasets than to the FIA plots (Fig. 7).

**Table 4:** Confusion matrix illustrating the number of pixels correctly and incorrectly classified in the various height classes in the target and imputed datasets.

		Target					
		3	8	18	38	70	accuracy
Imputed	**3**	**126,839,018**	491,213	9,242	577	0	**0.996**
	**8**	10,257,788	**334,363,687**	1,414,291	72,269	288	**0.966**
	**18**	4,434,215	1,848,028	**2,000,161,513**	3,368,066	5,831	**0.995**
	**38**	471,283	838,750	72,193	**353,795,222**	175,244	**0.996**
	**70**	32,855	20,755	1,231	4,892	**2,923,530**	**0.980**
	**accuracy**	**0.893**	**0.991**	**0.999**	**0.990**	**0.942**	**0.992**

**Fig. 9:**
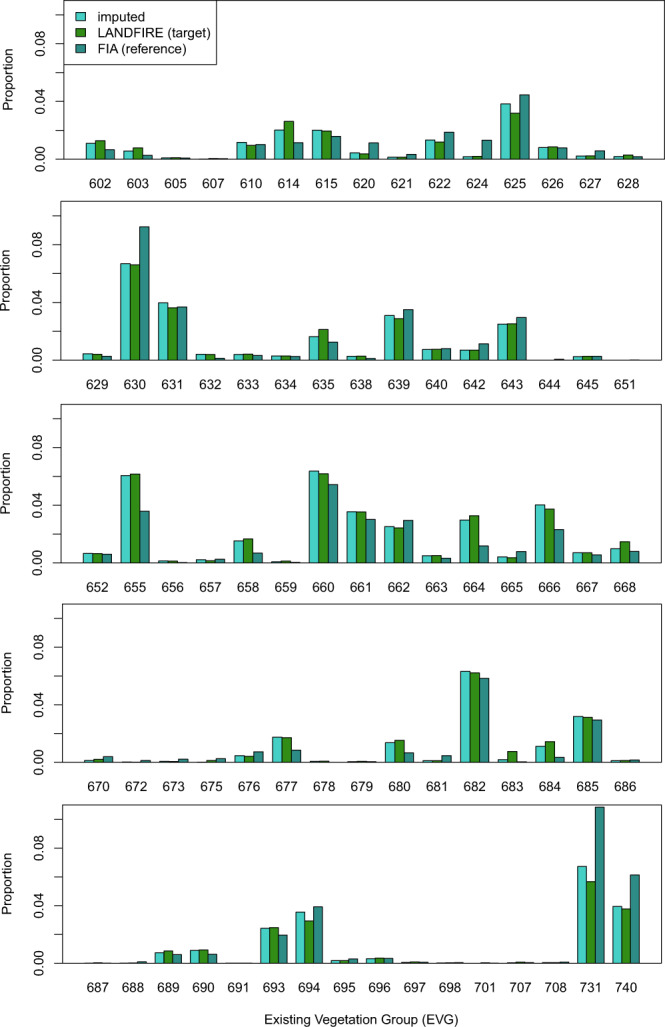
Proportion of FIA plots (reference), LANDFIRE pixels (target), and imputed pixels falling into each of the 76 EVG categories.

**Fig. 10:**
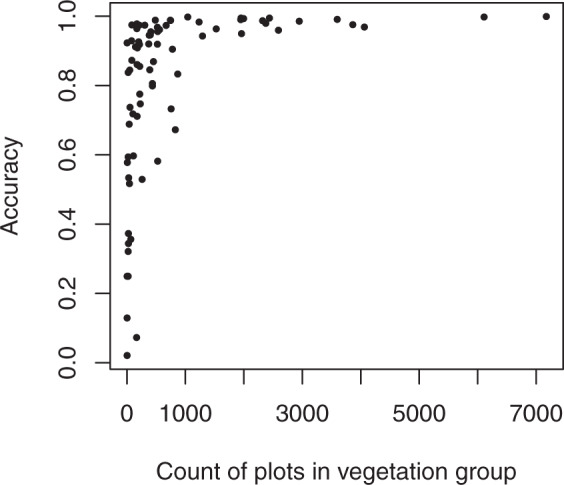
Producer’s accuracy by number of plots available for imputation in an EVG category.

At-pixel imputed EVG values matched those of the target data 93.0% of the time. For 43 of 76 categories, the producer’s accuracy exceeded 90%, and in 51 categories the user’s accuracy exceeded 90% (Online-only Table 1 and Supplementary Table 1). Of the 76 EVG categories, the lowest user’s accuracy for any given EVG was approximately 52% and the lowest producer’s accuracy was approximately 2% (Supplementary Table 1). In general, the proportion of the landscape in each EVG class was similar across the FIA plots, target data, and imputed dataset, however there were some exceptions (Fig. 9). Of the 76 EVG categories, 46 had fewer than 500 plots available for imputation, while 58 had fewer than 1000 (Fig. 10). Accuracy was generally lower where plot numbers were sparse (Fig. 10); however, this did not prevent the overall accuracy from being high.

**Fig. 11:**
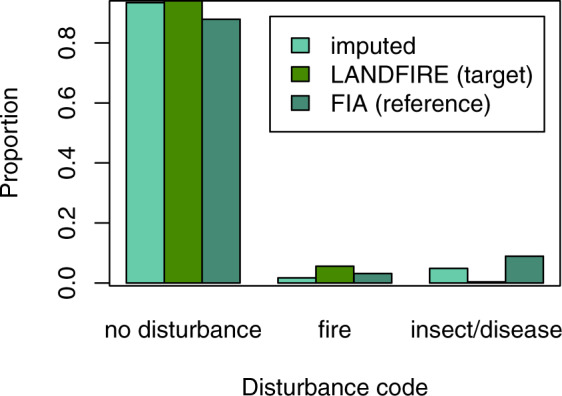
Proportion of FIA plots (reference), LANDFIRE pixels (target), and imputed pixels falling into each of the three disturbance categories.

**Fig. 12:**
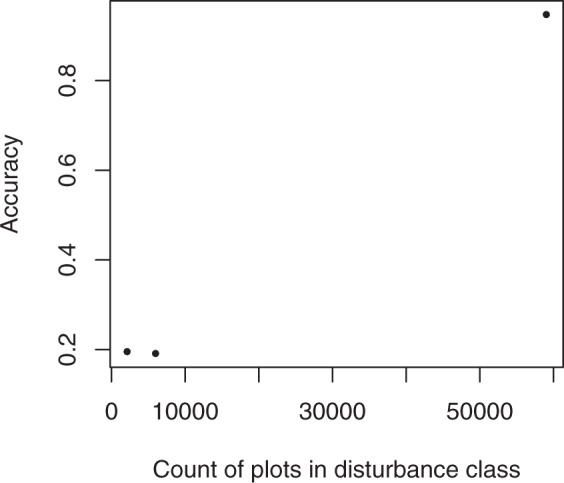
Producer’s accuracy by number of plots available for imputation in the three disturbance classes.

The imputed disturbance code (no disturbance, fire, or insect/disease) matched the LANDFIRE target dataset 90.3% of the time. However, accuracy in the disturbed categories was low, with a user’s accuracy of 64% in the fire class and 1% in the insect/disease class (Table 5). The low accuracies in the disturbed categories had little effect on the overall accuracy because the number of disturbed pixels is so much lower than the number of undisturbed pixels (only 5.6% of pixels were classified as burned and 0.4% as affected by insect/disease). Because the number of disturbed plots is also low, it’s difficult for random forests to match the disturbance class along with all the other variables, and disturbance wasn’t as important to the algorithm as vegetation group, cover, and height, as these were response variables. Accuracy was strongly affected by the count of plots in each disturbance class (Figs. 11 and 12), with high accuracies in the no-disturbance class (59,035 plots available for imputation) and low accuracies in the fire (2,111 plots) and insect/disease (5,995 plots) classes.

**Table 5:** Confusion matrix illustrating the number of pixels classified correctly and incorrectly in each disturbance class in the target and imputed datasets.

		Target			
		no disturbance	fire	insect/disease	accuracy
Imputed	no disturbance	**2,532,706,942**	114,511,377	8,522,868	**0.954**
	fire	17,207,086	**30,875,524**	191,143	**0.640**
	insect/disease	122,784,053	12,746,499	**2,056,489**	**0.015**
	**accuracy**	**0.948**	**0.195**	**0.191**	**0.903**

The proportion of the landscape in the various disturbance classes was similar across the FIA plot, target data, and imputed data; however, LANDFIRE may have underestimated the number of pixels in the insect/disease class, if the FIA plot data can be considered a reliable guide to the proportion of pixels affected (Fig. 11).

In future efforts, accuracy in the disturbance classes might be improved by 1) preferentially collecting more plot data in disturbed areas, 2) increasing the accuracy of mapping of insect/disease affected areas, or 3) including disturbance as a response variable. However, FIA’s plot locations are set in density, so FIA will not likely undertake additional collection. And if disturbance was included as a response variable without increasing the number of plots, the accuracy of the other response variables might suffer.

In short, the level of agreement between the target LANDFIRE rasters and the imputed dataset was very high (97.2% for cover, 99.2% for height, and 93% for vegetation group), suggesting the random forests methodology had a high level of success in identifying FIA plots with similar characteristics to the target data and imputing them at the scale of the continental US. Comparison of the FIA plot characteristics to the LANDFIRE target data identified a moderate level of accuracy in mapping cover within 10% (48.7%) and height within 5 m (70.2%) on at least one pixel within the plot’s radius. These differences could be caused by a number of factors, including: 1) errors in LANDFIRE’s mapping, 2) errors in measurement at FIA plot locations, 3) errors in the accuracy of FIA plot locations, and 4) errors in the estimation of cover and height at FIA plots resulting from the FVS routine (these may range from underestimation of approximately 2% where trees are clustered to overestimation of approximately 10% where trees are evenly spaced^30^). This moderate level of accuracy propagated to the imputed dataset (cover = 48.7% and height = 70.3%), since the target data values were used as predictor and response variables for each pixel in the random forests model. Errors in cover and height were most commonly in the adjacent bin (thus cover estimates were most frequently off by only 10–20%). We found that prediction of tree species had a higher accuracy (76.7%) than any of the three response variables. The imputed dataset has a similar proportion of the landscape in each cover, height, and vegetation group class as the FIA data (which should be representative of the landscape as a whole), suggesting that these variables can be reliably used in predictions at zone, regional, and national scales.

## Usage Notes

As noted above, the variables in the random forests algorithm were selected with the intention of optimizing the outputs for prediction of risk to carbon resources from wildland fire. The output is also likely quite suitable for predictions of biomass and basal area, including biomass removed by forest management projects such as fuel treatments to reduce fire hazard. The dataset may work well for such applications as species envelope modelling, among others. However, it has not been systematically validated for any of these uses at time of publication. Users are advised to assess the dataset for suitability of their project goals before proceeding.

The main output of the project is a map of imputed FIA plot IDs on a 30 × 30m raster grid, the TreeMap 2014. The utility of the dataset lies in its ability to be linked to databases that contain the FIA plot characteristics. We have provided an accompanying database in both Access and text format that lists inventory year and state for each plot, as well as for each tree, its status (live or dead), diameter, height, number of trees per acre conversion factor, etc. The raster and tables are available in the US Department of Agriculture Research Data Archive^15^. However, many more plot characteristics can be accessed via the FIA DataMart^11^, which serves the data by state in a variety of formats including HTML, XLS, SQLITE, and CSV. Specifically, to perform the linkage, users can leverage either of two columns present in the TreeMap raster at “\Data\national_c2014_tree_list.tif”: the tree list identifier (“tl_id”) field in the raster corresponds to a unique FIA sequence number (“CN”) also given in the raster’s attribute table. The “tl_id” and “CN” fields are also present in the accompanying tables at “\Data\trees_CONUS_5_15_2019.mdb\Tree_table_CONUS” or “\Data\Tree_table_CONUS.txt”. The sequence numbers also appear in the tables in the FIA DataMart (note that the sequence number has the attribute name “CN” in FIA’s “PLOT” table and “PLT_CN” in all other tables). The sequence number signifies a single visit to a plot; if the same plot is revisited it will have a new CN. All plot CNs utilized in this analysis were 100% forested, physically located within the boundaries of CONUS, and were obtained by MOU from FIA in December of 2012.

### Supplementary information

Supplementary Table 1

## Data Availability

Code was written in R and Python for the purposes of this project. The arcpy module was run in the Python version that accompanies ArcGIS 10.5.1, which is the Python IDLE 2.7.13. Code is available at https://github.com/USDAForestService/TreeMap2014_scripts. The code: 1)       prepared the target data rasters (script names: “reclass_Landfire_disturbance_rasters_for_tree_list.py” and “write_EVG_remap_files.r”) 2)      performed the random forests imputation, using the R package yaimpute (script name: “yai-parallel_v02202019-final-Yes-disturbance_z1.r”) 3)     validated the output grid of imputed plot IDs by comparing it to FIA plots measured in 2014 and to the target rasters (script names: “national_validation_plots_Landfire.py” and “national_validation_plots_Landfire_step2.r”) 4)         compared the imputed raster to the target rasters as a measure of imputation accuracy (script name: “analyze_national_tree_list_output.r”)

## Online-only Tables

**Online-only Table 1:** EVG numeric code and corresponding vegetation group name.

EVG code	EVG name
602	Aspen Forest, Woodland, and Parkland
603	Aspen-Mixed Conifer Forest and Woodland
605	Bigtooth Maple Woodland
607	Chaparral
610	Western Conifer-Oak Forest and Woodland
614	Douglas-fir Forest and Woodland
615	Douglas-fir-Western Hemlock Forest and Woodland
620	Juniper Woodland and Savanna
621	Limber Pine Woodland
622	Lodgepole Pine Forest and Woodland
624	Mesquite Woodland and Scrub
625	Douglas-fir-Ponderosa Pine-Lodgepole Pine Forest and Woodland
626	Mixed Broadleaf Evergreen Forest and Woodland
627	Mountain Hemlock Forest and Woodland
628	Mountain Mahogany Woodland and Shrubland
629	Western Oak Woodland and Savanna
630	Pinyon-Juniper Woodland
631	Ponderosa Pine Forest, Woodland and Savanna
632	Red Alder Forest and Woodland
633	Red Fir Forest and Woodland
634	Redwood Forest and Woodland
635	Western Riparian Woodland and Shrubland
638	Sitka Spruce Forest
639	Spruce-Fir Forest and Woodland
640	Whitebark Pine Woodland and Parkland
642	Western Hemlock-Silver Fir Forest
643	Douglas-fir-Grand Fir-White Fir Forest and Woodland
644	Western Larch Forest and Woodland
645	Western Red-cedar-Western Hemlock Forest
651	Alpine-Subalpine Barrens
652	Aspen-Birch Forest
655	Beech-Maple-Basswood Forest
656	Texas Live Oak
657	Cypress
658	Coastal Plain Oak Forest
659	Bur Oak Woodland and Savanna
660	White Oak-Red Oak-Hickory Forest and Woodland
661	Chestnut Oak Forest and Woodland
662	Post Oak Woodland and Savanna
663	Black Oak Woodland and Savanna
664	Chestnut Oak-Virginia Pine Forest and Woodland
665	Red Pine-White Pine Forest and Woodland
666	Eastern Floodplain Forests
667	White Oak-Beech Forest and Woodland
668	Eastern Small Stream Riparian Forests
670	Glades and Barrens
672	Hammocks
673	Hardwood Flatwoods
675	Inland Marshes and Prairies
676	Jack Pine Forest
677	Longleaf Pine Woodland
678	Mangrove
679	Maritime Forest
680	Sweetgum-Water Oak Forest
681	Montane Oak Forest
682	Yellow Birch-Sugar Maple Forest
683	Peatland Forests
684	Pine Flatwoods
685	Pine-Hemlock-Hardwood Forest
686	Pitch Pine Woodlands
687	Pocosin
688	Eastern Prairies and Barrens
689	Shortleaf Pine Woodland
690	Shortleaf Pine-Oak Forest and Woodland
691	Southern Scrub Oak
693	Spruce-Fir-Hardwood Forest
694	Atlantic Swamp Forests
695	Virginia Pine Forest
696	Juniper-Oak
697	Loblolly Pine Forest and Woodland
698	Loblolly Pine-Slash Pine Forest and Woodland
701	Introduced Riparian Vegetation
707	Introduced Upland Vegetation-Treed
708	Introduced Woody Wetland Vegetation
731	Managed Tree Plantation
740	Ruderal Forest

**Online-only Table 2:** Reference data sources and fields in FIA data used for each variable.

Category	Data Layer/variable name	Data URL or source
Vegetation	Existing Vegetation Cover (EVC)	Calculated from FIA plot data using “StrClass” keyword in the Forest Vegetation Simulator;^22^ cover calculations convey the percent of ground area covered directly by the crowns of trees, corrected to avoid overestimation due to crown overlap
	Existing Vegetation Height (EVH)	Calculated from FIA plot data using “StrClass” keyword in the Forest Vegetation Simulator;^22^ corresponds to the height of the top stratum, which was computed by averaging the height of the nine trees centered around the 70^th^ percentile tree
	Existing Vegetation Group (EVG)	Assigned from FIA plot data using LANDFIRE’s AutoKey process or in limited cases a manual classification
Topography	Aspect	ASPECT field in CONDITION table of FIA data, corresponding to the direction of slope in degrees for the majority of the condition^4^
	Slope	SLOPE field in CONDITION table of FIA data, corresponding to the percent slope of the condition^4^
	Elevation	ELEVATION field in the PLOT table of FIA data, corresponding to distance above sea level (the figure may be rounded to the nearest 10-foot or 100-foot category in some work units)^4^
Biophysical	Photosynthetically active radiation	Obtained via overlay of plot coordinates with LANDFIRE raster
	Precipitation	“
	Relative humidity	“
	Maximum temperature	“
	Minimum temperature	“
	Vapor pressure deficit	“
Disturbance	Disturbance code	Disturbance Code 1 (DSTRBCD1) in the CONDITION table of the FIA data. The Disturbance Code 1 indicates the type of disturbance, if one occurred since the last measurement or within the previous 5 years for new plots^4^
	Disturbance year	Disturbance Year 1 (DSTRBYR1) in the CONDITION table of the FIA data. The Disturbance Year 1 indicates the year in which the disturbance is estimated to have occurred^4^
Location	Latitude	Via Memorandum of Cooperation with FIA
	Longitude	“
